# Editorial: Multidisciplinary aspects and performance in racket sports-volume III

**DOI:** 10.3389/fpsyg.2026.1907776

**Published:** 2026-07-22

**Authors:** Jesus Ramón-Llin, Bernardino J. Sánchez-Alcaraz, Goran Vuckovic, Rafael Martínez-Gallego

**Affiliations:** 1 University of Valencia, Valencia, Spain; 2 University of Murcia, Murcia, Spain; 3 University of Ljubljana, Ljubljana, Slovenia

**Keywords:** badminton, padel, pickleball, table tennis, tennis

An interdisciplinary approach integrating exercise physiology, biomechanics, sport psychology, and nutrition has become essential for optimizing athletic performance (Weinberg and Gould, 2024; Jeukendrup and Gleeson, 2019). Combining knowledge from these different fields allows practitioners and researchers to better understand the complex factors associated with sporting success and to develop more comprehensive performance-enhancement strategies (Kraemer and Fleck, 2007). This holistic perspective not only contributes to improvements in physical qualities such as strength and endurance (Zatsiorsky et al., 2020), but also supports psychological components including motivation, emotional control, and stress management, ultimately fostering sustainable and high-level sport performance (Moran, 2013).

Thus, in this Research Topic of Frontiers in Psychology, titled “*Multidisciplinary Aspects and Performance in Racket Sports III*” racket sports continue to experience remarkable global expansion, not only in terms of participation but also regarding scientific interest. Tennis, padel, badminton, pickleball, and table tennis are increasingly recognized as multidisciplinary contexts where physiological, psychological, cognitive, technical, and social variables interact dynamically. The Research Topic of studies gathered in this Research Topic provides an updated and diverse overview of current research trends in racket sports science, emphasizing athlete development, wellbeing, tactical behavior, cognitive functioning, and training optimization across different populations and competitive levels.

One of the most evident themes emerging from these investigations is the growing relevance of psychological and cognitive factors in performance and participation. Traditionally, racket sports research focused heavily on physiological demands and biomechanical efficiency. However, recent evidence increasingly highlights the importance of mental processes, emotional regulation, motivation, and perceptual-cognitive skills.

In this regard, the study by Karakullukcu et al. explored the relationship between leisure involvement and re-participation intention among recreational tennis players. Their findings demonstrated that players with higher levels of involvement showed stronger intentions to continue participating in tennis activities. This research reinforces the role of psychological engagement as a determinant of long-term adherence to physical activity, particularly in recreational settings where enjoyment and intrinsic motivation are central components.

Similarly, Lauxtermann and Stubbs provided a systematic review examining padel and pickleball in relation to wellbeing and mental health outcomes. Their review revealed that pickleball participation is associated with increased life satisfaction, happiness, social integration, and reduced depressive symptoms, especially among older adults. In contrast, padel research has primarily focused on competitive psychological variables such as anxiety, self-confidence, and mental fatigue. Importantly, the review highlights the accessibility and social nature of emerging racket sports, positioning them as valuable tools for promoting public health and psychological wellbeing across populations.

The influence of mental fatigue on sports performance was further examined by Öztürker et al., who investigated its effects on attention and tennis groundstroke accuracy. Their results demonstrated that acute mental fatigue significantly impaired attentional performance and stroke targeting precision. These findings contribute to the growing body of evidence suggesting that cognitive exhaustion negatively affects technical execution in open-skill sports. From a practical perspective, coaches and sport psychologists should consider cognitive load management as an integral component of training design and competition preparation.

Cognitive and perceptual expertise was also addressed by Yufei and Ningning, who analyzed the relationship between spatial working memory and offensive tactical decision-making in tennis players. Expert athletes demonstrated superior visual search strategies, faster reaction times, and more efficient information processing compared with novice players. Furthermore, players with higher spatial working memory capacity showed more advanced gaze behaviors during tactical anticipation tasks. This study reinforces the idea that elite performance in racket sports depends not only on physical capacities but also on sophisticated perceptual-cognitive mechanisms that allow athletes to interpret rapidly changing competitive environments.

Technical and tactical analysis represented another major research direction across the included studies. Zhao et al. investigated technical-tactical diversity in elite tennis using entropy-based analyses during Grand Slam competitions. Their findings suggested that greater tactical diversity does not necessarily guarantee success. Instead, stability and effectiveness in technical execution appeared more relevant to match outcomes. Additionally, gender-related differences in tactical diversity were identified, providing meaningful insights for individualized coaching strategies at the elite level.

In table tennis, Xu et al. examined diagnostic index systems in the ABS plastic ball era. Their research proposed a refined five-segment model to better capture the technical-tactical dynamics of contemporary table tennis. Particularly noteworthy was the increased importance of connecting phases and transitional strokes between offensive and defensive actions. This work highlights how rule and equipment changes continuously reshape tactical demands, requiring ongoing adaptation in performance analysis methodologies.

Likewise, Zhao et al. compared temporal and technical-tactical characteristics in badminton singles under different competition formats. Their results showed that the newer 11-point relay system shortened overall match duration while maintaining rally intensity. This format appears to reduce physical load but simultaneously increases psychological pressure due to the importance of each rally. These findings have practical implications for competition design, coaching preparation, and athlete conditioning in youth badminton. Another relevant contribution was provided by Paulauskas et al., who compared the performance demands and game characteristics of doubles matches in padel and tennis. Their findings highlighted significant differences between both racket sports in variables related to external load, stroke distribution, rally structure, and movement patterns. Specifically, padel was characterized by longer rallies, a higher frequency of volleys, and lower-intensity movement profiles, whereas tennis doubles involved greater use of high-speed actions and more aggressive baseline play. These results underline the unique tactical and physical demands of each discipline and reinforce the importance of designing sport-specific training methodologies that reflect the distinct temporal and technical characteristics of padel and tennis competition.

Research on athlete development and youth training also occupied a central place in this Research Topic. Oliveira et al. analyzed the effects of a 6-week intervention program on young tennis players using a cluster analysis approach. While overall improvements were observed in agility, sprint performance, and International Tennis Number rankings, substantial inter-individual variability emerged. Some athletes improved their classification while others regressed, emphasizing the necessity of individualized training programs that account for developmental differences and unique adaptation responses.

The importance of biological maturation was further explored by Rodríguez-Campos et al., who investigated the effects of ball type and maturity status on competition load in under-10 tennis players. Interestingly, the use of low-compression green balls did not substantially increase physical demands compared with standard balls. Instead, biological maturation emerged as the strongest determinant of match load characteristics. These findings support the pedagogical use of adapted equipment while simultaneously emphasizing the need for maturity-based approaches to youth training and competition planning.

Youth athlete profiling was also examined in badminton by Winata et al. Their study revealed significant age- and sex-related differences in anthropometric characteristics and anaerobic capacities among highly trained junior badminton players. Male players generally displayed superior sprint and power performances, whereas female athletes showed different developmental trajectories. Such findings contribute valuable normative information for talent identification and long-term athlete development programs.

Another prominent topic in this Research Topic concerns the educational and psychosocial value of racket sports. Wang et al. demonstrated that a 16-week table tennis intervention significantly reduced short-video addiction among Chinese university students. Importantly, cognitive bias reduction and improved self-control mediated this relationship. This study illustrates how sport participation can serve as an effective behavioral intervention strategy in the digital age, where excessive screen exposure increasingly affects young populations.

In a related educational context, Wang et al. investigated the influence of systematic table tennis training on the Big Five personality traits among primary school students. Their findings revealed improvements in openness, agreeableness, extraversion, and emotional stability following the intervention. These results suggest that structured sport participation can positively contribute to personality development during childhood, reinforcing the educational value of physical activity beyond purely physical benefits.

Motor learning and pedagogical methodologies were explored by Qu et al., who analyzed contextual interference effects during volleyball serve acquisition. Although not strictly focused on racket sports, this study contributes important insights into skill learning processes that are highly transferable to racket sport coaching. Their results indicated that block practice enhanced initial acquisition, random practice improved transfer, and mixed practice optimized long-term retention. Such findings reinforce the importance of varied and strategically structured practice environments in developing adaptable athletes.

Collectively, these studies illustrate the increasingly interdisciplinary nature of racket sports science. Performance is no longer understood as the product of isolated physiological capacities but rather as the interaction between cognitive functioning, psychological wellbeing, tactical behavior, developmental processes, social engagement, and contextual factors.

Several broader implications emerge from this body of work. First, individualized approaches are becoming essential in athlete development. Whether considering maturation status, cognitive characteristics, tactical preferences, or psychological profiles, one-size-fits-all models appear increasingly inadequate. Second, the psychological and social dimensions of racket sports deserve greater attention, particularly given the growing evidence supporting their contributions to mental health, wellbeing, and behavioral regulation. Third, technological and methodological advances in performance analysis are enabling more sophisticated understandings of tactical complexity and athlete behavior.

Future research should continue integrating multidisciplinary perspectives while expanding methodological rigor through longitudinal designs, ecological assessments, and more diverse populations. Greater attention should also be devoted to female athletes, recreational participants, and underrepresented age groups. Additionally, emerging racket sports such as padel and pickleball offer promising opportunities for investigating lifelong participation, social wellbeing, and sustainable physical activity promotion.
